# Mutations in the cardiac troponin T gene show various prognoses in Japanese patients with hypertrophic cardiomyopathy

**DOI:** 10.1007/s00380-013-0332-3

**Published:** 2013-03-14

**Authors:** Etsuko Fujita, Toshio Nakanishi, Tsutomu Nishizawa, Nobuhisa Hagiwara, Rumiko Matsuoka

**Affiliations:** 1 Department of Cardiology, Tokyo Women’s Medical University, Tokyo, Japan; 2 Department of Pediatric Cardiology, Tokyo Women’s Medical University, Tokyo, Japan; 3 International Research and Educational Institute for Integrated Medical Sciences (IREIMS), Tokyo Women’s Medical University, Tokyo, Japan; 4 Division of Genomic Medicine, Institute of Advanced Biomedical Engineering and Science, Graduate School of Medicine, Tokyo Women’s Medical University, Tokyo, Japan; 5 International Center for Molecular, Cellular, and Immunological Research (IMCIR), Tokyo Women’s Medical University, 8-1 Kawada-cho, Shinjuku-ku, Tokyo, 162-8666 Japan

**Keywords:** Familial hypertrophic cardiomyopathy, *TNNT2* gene, Mutation, Phenotype–genotype

## Abstract

Hypertrophic cardiomyopathy (HCM) is an autosomal dominant disorder resulting from mutations in genes for at least 15 various sarcomere-related proteins including cardiac β-myosin heavy chain, cardiac myosin-binding protein C, and cardiac troponin T. The troponin T gene (*TNNT2*) mutation has the third incidence of familial HCM, and the genotype–phenotype correlation of this gene still remains insufficient in Japanese familial HCM. Therefore, in the present study, we focused on screening the *TNNT2* mutation in 173 unrelated Japanese patients with familial HCM, and found three reported mutations and a new mutation of *TNNT2* in 11 individuals from four families. In these families, two individuals from one family had double mutations, Arg130Cys and Phe110Ile, six individuals from two other families had an Arg92Trp mutation, and one individual of another family had a new mutation, Ile79Thr, of *TNNT2*. The phenotype of each family was often different from reported cases, even if they had the same genetic mutation. In addition, families with the same genetic mutation showed a similar trend in the phenotype, but it was not exactly the same. However, sudden death in youth was observed in all of these families. Although the type of genetic mutation is not useful for predicting prognosis in HCM, the possibility of sudden cardiac death remains. Therefore, the prognosis of individuals bearing the *TNNT2* mutation with familial HCM should be more carefully observed from birth.

## Introduction

Hypertrophic cardiomyopathy (HCM) is characterized by left and/or right ventricular hypertrophy, with predominant involvement of the interventricular septum in the absence of other causes of hypertrophy, such as hypertension, valvular heart disease, or metabolic disease [1–7].

Familial HCM is an autosomal dominant disorder caused by mutations in genes that encode sarcomere proteins. It has been reported that at least 15 genes are implicated in 55–70 % of HCM, and the major genes causing HCM include cardiac β-myosin heavy chain (*MYH7*), α-tropomyosin, cardiac troponin T (*TNNT2*), cardiac myosin-binding protein C (*MYBPC3*), cardiac troponin I (*TNNI3*), cardiac myosin regulatory light chains (*MYL2*), and cardiac myosin essential light chains (*MYL3*) [1, 4, 5, 8–11].

Cardiac troponin T is a thin-filament protein that takes part in muscle contraction. The troponin complex on the actin filament regulates the force and velocity of muscle contraction. Troponin C functions as a calcium receptor while troponin I inhibits adenosine triphosphatase (ATPase) activity when bound to actin. Troponin T fixes the troponin group to tropomyosin. During relaxation, the troponin group is bound to actin and tropomyosin, blocking the interaction of myosin and actin [12].

*TNNT2* was mapped to chromosome 1q32. Mutations of *TNNT2* were thought to account for approximately 15 % of familial HCM, with most missense mutations located in exons 8–16 [3], and were associated with a particularly severe form of the disease characterized by a poor overall prognosis with a high incidence of sudden death despite only mild left ventricular hypertrophy (LVH) [1–4]. Most *TNNT2* mutations of familial HCM alter the contractile properties of cardiac muscle, especially the Ca^2+^ sensitivity of force development and ATPase activity in vitro and in vivo [13–17].

Previous reports have suggested that there is a more consistent relationship between certain genetic mutations and clinical outcome, allowing for the classification of “benign” and “malignant” mutations [18–30]. For example, a favorable prognosis has been reported in patients with a Phe110Ile mutation of the *TNNT2* mutation (in 16 individuals of 6 Japanese families) [19, 20]. From these data, genetic analysis and determination of genotype were thought to be important for assisting with patient management.

However, these findings were based on limited experience; to date, only 30 different mutations have been identified in *TNNT2* [9–11, 18]. In addition to this genetic diversity, the phenotypic expression of these mutations varies considerably, ranging from asymptomatic individuals with a normal life expectancy to those with sudden cardiac death or the need for an early heart transplant. Clinical parameters such as the degree of LVH, the presence or absence of a left ventricular outflow tract gradient, and electrophysiology testing have not been predictive markers of poor prognosis [1–5]. More recently, it was reported that late gadolinium enhancement with cardiac magnetic resonance can be a predictive marker of the ventricular arrhythmia and poor prognosis in HCM [31].

In recent years, mutation-specific risk stratification was considered to be not possible, but genetic test-based risk stratification seemed to be clinically informative [32].

The *TNNT2* mutation has the third-ranked incidence of familial HCM, and the genotype–phenotype correlation of this gene still remains insufficient in Japanese familial HCM. Therefore, in the present study we focused on screening the *TNNT2* mutation.

## Patients and methods

### Subjects

We genetically evaluated 173 patients (101 men and 72 women; 0–79 years old, median age 20 years) who were clinically diagnosed with familial HCM.

Pediatric patients with HCM were recruited from Tokyo Women’s Medical University. Written informed consent was obtained from all study subjects in accordance with the Declaration of Helsinki. If patients were younger than 16 years, informed consent was given by their guardians. We assessed each patient by taking their history and performing a physical examination, and reviewed their medical records. All assessments were done with the approval of the Ethics Committees of Tokyo Women’s Medical University.

The diagnosis of HCM was determined through clinical evaluation, chest radiography, electrocardiography, echocardiography, and cardiac catheterization based on current international consensus criteria.

Diseases of the heart, hypertension, valvular heart disease, or metabolic disease attributable to HCM were excluded from this study by pediatric cardiologists.

### Mutation screenings

Genomic DNA was prepared from peripheral blood lymphocytes or lymphoblast cell lines transformed by the Epstein–Barr virus, as described previously [33]. *TNNT2* coding regions and exon–intron boundaries, including regions approximately 30–100 bp upstream and downstream from the exons, were amplified from genomic DNA using primers, as described in previous reports [34, 35]. Genomic DNA (50 ng) was amplified through the use of primers designed from flanking intron sequences (Table 1).

**Table 1:** Primers used for amplification of fragments from *TNNT2*

Exon	Forward (5′–3′)	Reverse (5′–3′)
2	GAGCTCTTCTGAGGAAGGCA	CTACCCAGAATCCGAGGGAC
3 and 4	CAGGGCAGCGTGGACTCCC	CCCAGGGCTCCCAGGATTT
5	CCATTCTCTGCTCTGGGTTC	GTGCACATGGGAAAGCTGTTCT
6	TAGGGCTTATCTGTGGGGAAGGC	CTTCCCTGGAAAGAGCACTG
7	GAAATGGAAATCCACAGGGA	TACTGCACCCCGTTCCATCA
8 and 9	CTCTAGGAAGGATCAGGGCCC	CTCACAAAAGGGATGGAGGA
10	GCGATGTCACCTTCTCCCTA	CACCGCACCCGGCCAATA
11	GGTTTCCAATCCTTTCCCCTAA	GCTGCAGTGGACACCTCATTC
12	GCCTTTGTCTTCCTGCCTTCTC	CAGCCAGCCCAATCTCTTCACT
13	ACAGGGAGGGGGCAATCTGGCC	CCCAGAGCAGATGCGGGCAGTG
14	ACTGGGTGCTGCCGTCTGGTC	AAGGGGGCTGTTGGGGAATAGG
15	CACTCAGCCCCCTTCTCC	AAGCTTCTCCGCCCCACATTTC
16	GGCACCCCAGTCCTACCC	GTCCCCCTCAACAGCACTTTT

Amplified products were purified using a MultiScreen polymerase chain reaction (PCR) plate (Millipore, Billerica, MA, USA) and directly sequenced using the ABI-PRISM BigDye-terminator cycle sequencing reaction kit and ABI 3130*xl* genetic analyzer (Applied Biosystems, Foster City, CA, USA). When a mutation was detected, we confirmed that it was not presenting 363 Japanese normal chromosomes by direct sequencing.

The PCR conditions of these genes were modified and fragments were analyzed by electrophoresis, as described previously [36, 37].

Proband patients bearing *TNNT2* mutations were additionally examined for reported HCM mutations, the coding regions of *MYH7*, *MYBPC3*, *TNNI3*, α-tropomyosin, *MLY2*, *MLY3*, and all exons, and a promoter region of the α-galactosidase A gene. Primer sequences and detailed PCR conditions for these additional analyses are available upon request. Reference sequences and single-nucleotide polymorphism information were obtained from the National Center for Biotechnology Information.

## Results

### Clinical report and DNA analysis

To investigate mutations of *TNNT2*, we performed DNA analysis in 173 unrelated Japanese patients with familial HCM. We identified three reported mutations and a new mutation of *TNNT2* in 11 individuals from four families (A, B, C, D) (Table 2).

**Table 2:** Clinical features of patients with *TNNT2* mutation in families A, B, C, and D

HCM individuals	A III-2	A III-1	A II-6	A II-6′ (III-2/mother)	B III-7	B III-2	B IV-3	C IV-3	C III-3	C II-3	D III-2
Exon	9 and 10	9 and 10	9	10	9	9	9	10	10	10	8
Nucleotide substitution	T328A and C388T	T328A and C388T	T328A	C388T	C274	C274	C274	C388	C388	C388	T236C
Amino substitution	P110I and R130C	P110I and R130C	P110I	R130C	R92W	R92W	R92W	R92W	R92W	R92W	I79T
Sex	F	M	M	F	F	M	M	F	F	F	F
Age (years)	12	14	55	46	35	41	2	42	15	80	25
Outcome (years)	12, Vf survivor	14, died suddenly									24, Vf survivor
Clinical diagnosis	HCM	HCM	CAVB, PMI	Normal	DHCM (HCM)	Abnormal ECG	HCM	HCM	HCM	HCM	HCM
Electrocardiogram	SR + CRBBB	SR + CRBBB, abnQ	PM rhythm (CAVB)	WNL	R-wave progression	Details unknown	RVH, deep Q	LVH, ST dep	Details unknown	Details unknown	ST dep, inv T
2D echocardiogram	LVH, ASH	LVH, ASH	LVH	WNL	LVH, apical hypertrophy	Details unknown	LVH	LVH, ASH	LVH	Details unknown	LVH, ASH
Cardiac catheterization	LV relaxation abnormalities	LV relaxation abnormalities	LV relaxation abnormalities	ND	ND	ND	ND	ND	ND	ND	ND

*HCM* hypertrophic cardiomyopathy, *HOCM* hypertrophic obstructive cardiomyopathy, *DHCM* dilated phase of HCM, *LVH* left ventricular hypertrophy, *Vf* ventricular fibrillation, *CAVB* complete atrioventricular block, *SR* sinus rhythm, *CRBBB* complete right bundle branch block, *abnQ* abnormal Q wave, *LVH* left ventricular hypertrophy, *RVH* right ventricular hypertrophy, *ST dep* ST depression, *inv T* inversion T, *PMI* pacemaker implantation, *ECG* electrocardiogram, *ASH* asymmetric septal hypertrophy, *APH* apical hypertrophy, *ND* not determined, *WNL* within normal limits

**Fig. 1:**
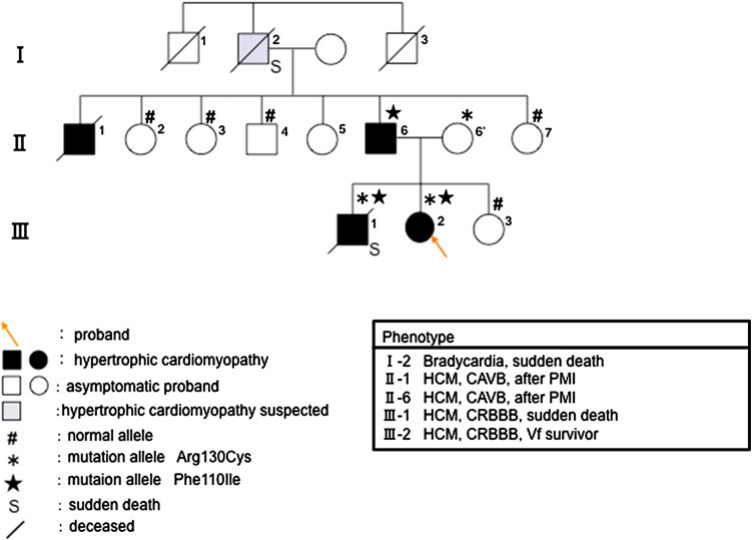
Pedigree and phenotype of Family A. Pedigree with three generations (*Roman Numerals*), the propositus is denoted by an *arrow*. Further clinical data for family members is detailed in the *table*. *HCM* hypertrophic cardiomyopathy, *CAVB* complete atrioventricular block, *PMI* pacemaker implantation, *CRBBB* complete right bundle-branch block, *Vf* ventricular fibrillation

In family A, all five members who carried or were suspected of having HCM also had arrhythmia. Four of them had blocks, including a complete atrioventricular block (CAVB) and complete right bundle branch block (CRBBB) (Fig. 1; II-1, 6, III-1, 2), and one had bradycardia. Two of them died suddenly (Fig. 1; I-2, III-1), and one was a ventricular fibrillation (Vf) survivor (Fig. 1; III-2). The proband (Fig. 1; III-2), aged 12 years, with sinus rhythm (SR) and a CRBBB, had an episode of Vf, and her elder brother (Fig. 1; III-1), aged 14, also with SR and a CRBBB, had died suddenly. Her father and her uncle had pacemakers implanted to treat complete AV blocks. Her mother and mother’s family had no symptoms or abnormal findings. The proband and her brother and father (Fig. 1; III-2, III-1, I-2) showed mild LVH (maximum wall thickness <20 mm). All three members underwent cardiac catheterization, and showed significant left ventricular relaxation abnormalities.

DNA analysis showed that the proband and her elder brother had the double reported mutations, Arg130Cys and Phe110Ile, of *TNNT2* (Fig. 1). Her father, who had arrhythmia and mild HCM, had the Phe110Ile mutation, and her mother (II-6′), who had no symptoms or abnormal findings, had the Arg130Cys mutation.

**Fig. 2:**
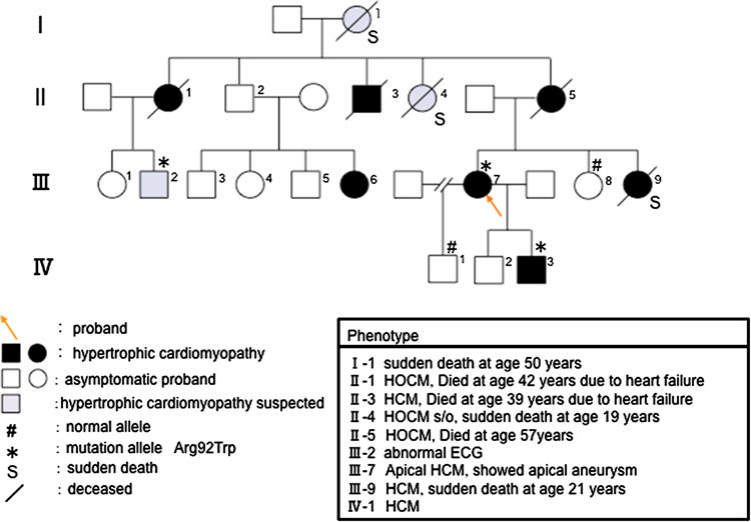
Pedigree and phenotype of Family B. Pedigree with four generations (*Roman Numerals*), the propositus is denoted by an *arrow*. Further clinical data for family members is detailed in the *table*. *HCM* hypertrophic cardiomyopathy, *HOCM* hypertrophic obstructive cardiomyopathy, *ECG* electrocardiogram

In family B, 3 of 10 members who carried or were suspected of having HCM were thought to have obstructive hypertrophic cardiomyopathy. Two female members (Fig. 2; II-4, III-9) died suddenly at a young age. The proband (Fig. 2; III-7) showed apical hypertrophy and apical aneurysm at a comparatively young age. Her aunt (Fig. 2; II-4), who had a heart murmur, was thought to have hypertrophic obstructive cardiomyopathy (HOCM), and died when she was 19 years old. Her younger sister (Fig. 2; III-9) did not show obstructive HCM, but died suddenly when she was 21 years old. Her grandmother (Fig. 2; I-1) died suddenly at the age of 50 years. DNA analysis showed that the proband (Fig. 2; III-7), her son (Fig. 2; IV-3), and her cousin (Fig. 2; III-2), who carried HCM, had an Arg92Trp mutation of *TNNT2*.

**Fig. 3:**
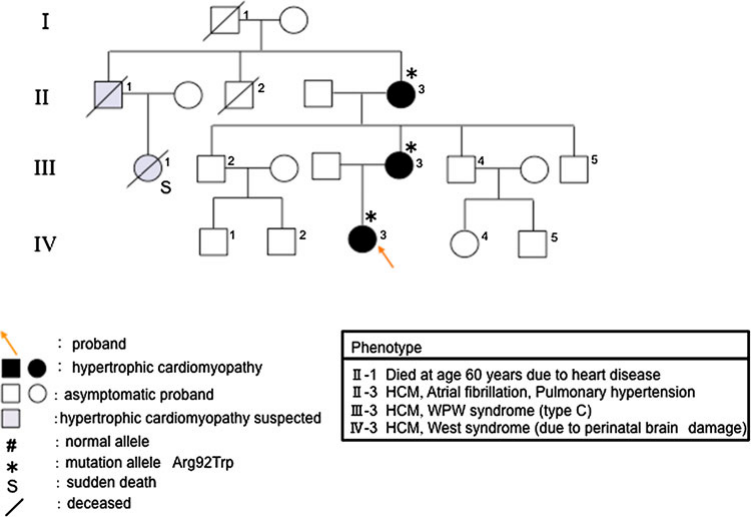
Pedigree and phenotype of Family C. Pedigree with four generations (*Roman Numerals*), the propositus is denoted by an *arrow*. Further clinical data for family members is detailed in the *table*. *HCM* hypertrophic cardiomyopathy, *WPW syndrome* Wolff-Parkinson-White syndrome

In family C, the proband (Fig. 3; IV-3), her mother (Fig. 3; III-3), and her grandmother (Fig. 3; II-3) showed mild cardiac hypertrophy (maximum wall thickness <20 mm) and asymmetric septal hypertrophy, and her myocardial hypertrophy had been gradually increasing. Her mother was suspected of having a shifting dilated phase by recent cardiac magnetic resonance imaging (MRI). The proband (Fig. 3; IV-3) had symptomatic West syndrome following perinatal brain damage, and was treated with corticotropin. DNA analysis showed that the proband (Fig. 3; IV-3) and her mother (Fig. 3; III-3) had the Arg92Trp mutation of *TNNT2*.

**Fig. 4:**
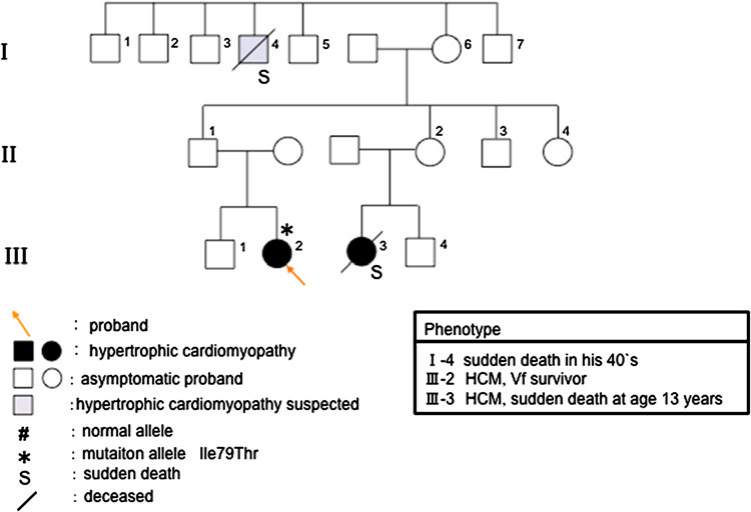
Pedigree and phenotype of Family D. Pedigree with four generations (*Roman Numerals*), the propositus is denoted by an *arrow*. Further clinical data for family members is detailed in the *table*. *HCM* hypertrophic cardiomyopathy, *Vf* ventricular fibrillation

**Fig. 5:**
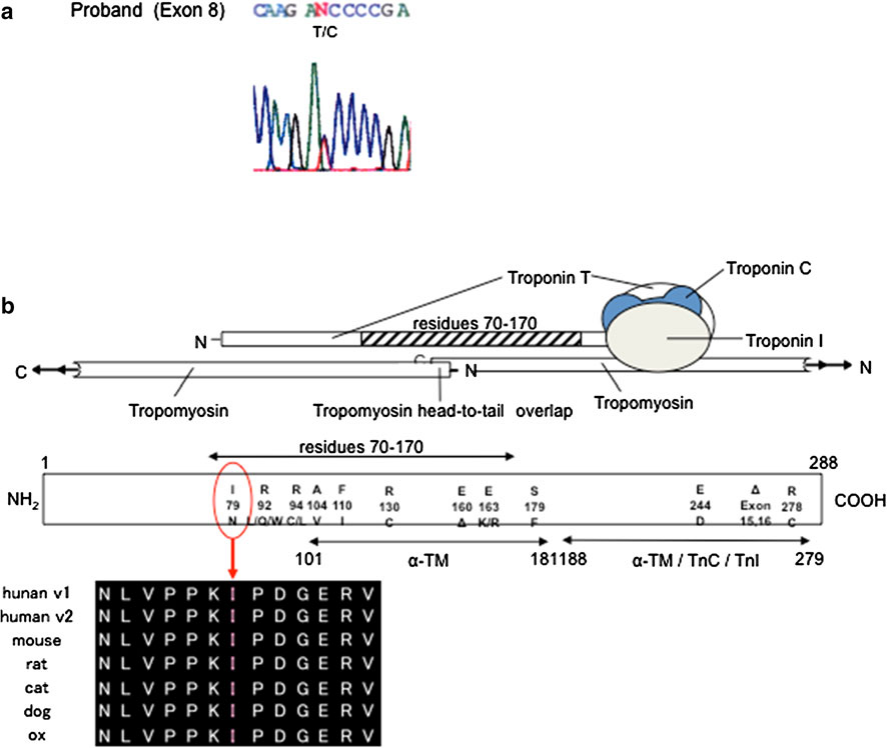
**a** The new Ile79Thr mutation of *TNNT2* (*Exon 8*). **b** The position of the 79th Ile codes for the binding site of tropomyosin. This domain is well preserved in mammals

**Fig. 6:**
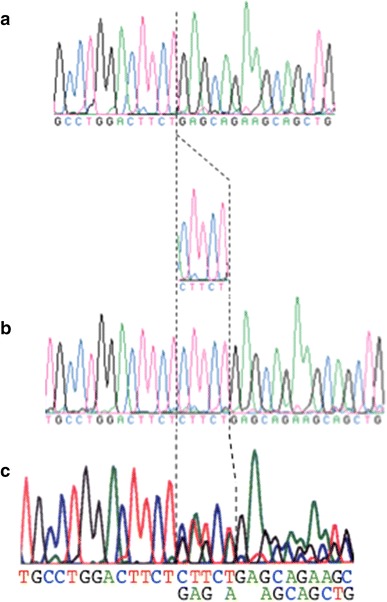
**a** The deletion/deletion (D/D) polymorphism. **b** The insertion/insertion (I/I) polymorphism. **c** The insertion/deletion (I/D) polymorphism

In family D, two of three members who carried or were suspected of having HCM died suddenly (Fig. 4; I-4, III-3), and one was a Vf survivor (Fig. 4; III-2). The proband (Fig. 4; III-2) had relatively strong cardiac hypertrophy (maximum wall thickness >20 mm), and cardiac standstill from ventricular fibrillation at the age of 24 years. Her cousin (Fig. 4; III-3) died suddenly at a young age (13 years), and her granduncle also died at the age of 40. DNA analysis shows that the proband had the 236T → C nucleotide alteration. This transition was observed in codon 79, and converted an isoleucine residue to a threonine residue (Fig. 5a). Ile79Thr occurred in conserved residues found in *TNNT2* orthologues of human, mouse, rat, cat, and ox (Fig. 5b). This mutation was not observed in 363 chromosomes from unaffected Japanese populations. The proband (Fig. 4; III-2) also had a 5-bp (CTTCT) deletion/deletion polymorphism of intron 3 of *TNNT2* (Fig. 6a).

To clarify the clinical importance of this polymorphism, we performed genetic analysis in 47 HCM patients from 173 unrelated Japanese patients with familial HCM. In these 47 patients, 24 had the deletion/deletion polymorphism (51 %, 14 men and 10 women; 0–72 years old, median age 30 years old). Five of the 24 (21 %) patients who had the deletion/deletion polymorphism were diagnosed with apical HCM or HOCM. In 6 of the 10 (60 %) with available data of echocardiography among these 24 patients, the maximum wall thickness was more than 30 mm. On the other hand, in the 47 HCM patients, the remaining 23 did not have the deletion/deletion polymorphism, but had the deletion/insertion or insertion/insertion polymorphism (49 %, 15 men and 8 women; 0–76 years old, median age 18 years). Two of the 23 (9 %) patients were diagnosed with apical HCM, and there were no HOCM patients. Two of the seven (29 %) with available data of echocardiography among these 23 patients showed the maximum wall thickness of more than 30 mm.

## Discussion

Previous studies have reported familial HCM cases where a consistent relationship between certain genetic mutations and clinical outcome was observed, allowing for the classification of “benign” and “malignant” mutations [18–30].

However, in recent years it was considered that the specific gene mutation cannot be the sole factor that dictates clinical phenotype. Since around 1 %–2 % of all patients with HCM had a “benign” or “malignant” mutation, mutation type was not seen to be clinically useful in predicting prognosis in HCM given this very low incidence rate [32, 38–40]. In addition, it was reported that mutation of a sarcomeric protein gene can cause RCM, HCM, and DCM within the same family, and patients with a benign mutation experienced a very serious clinical course [41, 42].

In the present study, the phenotype of each family was often different from reported cases, even if they had the same genetic mutation. In addition, families with the same genetic mutation showed a similar trend in the phenotype, but it was not exactly the same. However, sudden death in youth was observed in all of these families. On the other hand, there seemed to be a family with a “malignant” mutation, which did not show the phenotype of HCM or a family history of sudden death.

For example, in family A, two *TNNT2* mutations were found, the Arg130 Cys and Phe110Ile mutations. The Arg130 Cys mutation has been reported by a Chinese research group [21], whose clinical features had an early onset, syncope, sudden death in youth, heart failure, and arrhythmia as atrial fibrillation. The Phe110Ile mutation has been reported to show a favorable prognosis, and have comparatively slight hypertrophy or apical hypertrophy (in 16 individuals of 6 Japanese Families) [19, 20]. Both of these reported pedigrees are Asian.

In family A, whereas the proband and her elder brother, who carried these two mutations, showed similar reported phenotypes of the Arg130 Cys mutation [21], her mother and mother’s family members, who had the Arg130Cys mutation, showed no abnormal clinical features such as cardiac hypertrophy, sudden death, or abnormal electrocardiograms. Furthermore, her father and father’s family members, who had the Phe110Ile mutation, showed atrioventricular blocks, and these phenotypes have not been reported.

In family B, an Arg92Trp mutation of *TNNT2* was found. The mutation showed comparatively slight cardiac hypertrophy or a high incidence of sudden death in males (19 individuals of two mixed racial families) [22–24]. However, the proband and her family, who carried HCM, showed relatively severe cardiac hypertrophy, with a high incidence of sudden death in females.

In family C, an Arg92Trp mutation was also found. The proband and her family, who carried HCM, showed mild cardiac hypertrophy and asymmetric septal hypertrophy. The proband’s myocardial hypertrophy had been gradually increasing, and her mother had a suspected shift to the dilated phase.

In family D, the proband carried the new mutation Ile79Thr of *TNNT2*. She survived following an episode of Vf, and her cousin died suddenly at the age of 13 years. Previously, a mutation of the 79 residue, the Ile79Asn mutation, had been reported [24, 25], and this mutation also showed a poor prognosis. In our mutated case, even if the amino acid mutation (Ile79Thr) was different from the reported case (Ile79Asn), the patient showed a malignant prognosis. In family D, a 5-base-pair (CTTCT) deletion/deletion (D/D) polymorphism in intron 3 of the *TNNT2* was also found. It has been reported that this polymorphism had caused skipping of exon 4 of *TNNT2*, and that the deletion allele could be associated with a predisposition for prominent LVH [42]. Although it was in a very limited range, from our genetic study on this polymorphism we gained the impression that a patient carrying the *TNNT2* deletion/deletion polymorphism had a stronger tendency toward hypertrophy. To conduct further analysis, further examination including new cases is required.

We considered that, at least in Japanese familial HCM, only the type of genetic mutation of *TNNT2* did not seem useful in distinguishing the prognosis. However, if mutations were found, there was a risk of sudden death in youth. Therefore, regardless of the type of genetic mutation, it would be more important to observe the patient in detail from birth in each lineage.

The development of a “case-based” method would be useful to treat and help each individual, and more attention needs to be paid toward searching for the modifier and environmental factors including diet, lifestyle, exercise, and the modification gene and polymorphism.
